# The Medical Society of the State of New York

**Published:** 1900-03

**Authors:** 


A Monthly Review of Medicine and Surgery.

*EDITOR:*

WILLIAM WARREN POTTER, M. D.

All communications, whether of a literary or business nature, books for review and exchanges should be addressed to the editor: 284 FRANKLIN STREET, BUFFALO, N. Y.

VOL. XXXIX.—LV. MARCH, 1900. NO. 8.

### THE MEDICAL SOCIETY OF THE STATE OF NEW YORK.

THE ninety-fourth annual meeting of this society was held in the city hall, Albany, January 30, 31 and February 1, 1900. From a scientific standpoint this meeting will take rank among the foremost in the history of the society, while in regard to attendance and *esprit de corps* it probably outclasses any meeting held in later years. On the first and second days the spacious council chamber was much too small for the large gathering. Some of this difficulty may be obviated in future if the treasurer, trunk line agent and credential committee were given a sepaiate room. There are rooms nearby that could be utilised for this purpose, thereby relieving the meeting room of considerable pressure.

The president, Dr. Willis G. Macdonald, in his inaugural address referred to the general trend of medical affairs throughout the state, citing the satisfactory condition of higher medical education, the necessity for fixing more clearly the legal definition of the term "practice of medicine," so that all species of quackery may be controlled by our present medical practice act; the necessity for state control of tuberculosis; medical expert witnesses; the dangers of the measure before congress to regulate vivisection in the District of Columbia; and the reorganisation and increase of the medical corps of the army.

Dr. Macdonald's analysis of the differences between this society and the American Medical Association is so clear that we quote at length from the address:

The events leading to the schism now existing between the American Medical Association and the Medical Society of the State of New York are too well understood to require any repetition in this body, nor would any reference to it be made but for the unofficial discussion by individual members of each society of a plan for reappıoachment. The principles for which each faction in the profession of New York State has striven so strenuously and honestly for twenty years have, under the influence of medical progress, become practically identical. There lingers yet but the memories of thrusts received in heated if not acrimonious debate, and of wrongs perpetrated in defense of false notions. Personally, after a critical investigation, I can find no difference in the ethical behavior of members of the American Medical Association and that of members of the Medical Society of the State of New York. In fact, some very worthy and distinguished physicians are members of both and reflect equal credit upon each society. It has occurred to many of us that honesty and good manners are not matters for legislation or codes, but rather the results of home training and education, and that physicians are well bred and honest because of education rather than codes. The present medical law of the State of New York has done more to remove sects in medicine and improve the ethical and scientific standing of physicians than all the codes since Hippocrates. It is this law, possibly somewhat modified, with some additional safeguards, that is the code of ethics of the Medical Society of the State of New York, and practically unifies the profession of this state. If the present requirements for the degree of doctor of medicine in New York State will not guarantee the ethical behavior of the candidate, no code will. His state certificate is his authority and you can decline to receive, resolve and expel until red in the face, yet sit with the same man on an official board of health the next day.

The cases are isolated indeed where the code of the American Medical Association has been invoked to discipline a member. Its most frequent use has been its employment to keep members of this society from participating in the meetings of the American Medical Association. Recently this policy of conciliation has led to the acceptance of members of the Medical Society of the State of New York as members of the American Medical Association without asking any questions or having the gentleman recommended by another. The practice seems to me not only questionable but undignified. The differences which led to the disruption have disappeared. There is nothing but the echoes of strife and a few personal ambitions. A solution of the problem might be simple. Let the American Medical Association invite the Medical Society of the State of New York to send delegates whose credentials will be received and they be admitted through the same door that closed in the faces of that distinguished delegation of New York physicians at St. Paul. Let them come in the front door of the convention with equal rights for all; in this way, I believe, can our differences be settled with dignity, and the Association made what it is not now, the representative of the united medical profession of the United States.

The reports of committees elicited the fact that during the year much work had been done especially by the committees on by-laws, legislation, and hygiene, the report of the latter by the chairman, Dr. H. R. Hopkins, of Buffalo, being one of the most important and valuable made to the society in years. (See p. 602.)

The discussion on state care of tuberculous patients held in the assembly chamber, Tuesday evening, January 30th, was timely and must have shown the legislators present, that the society is earnest and serious in its advocacy of state care, and is ready to do its share in the alleviation and stamping out of a disease which kills 13,000 inhabitants of the Empire State every year. Interesting papers on this subject were read by Drs. Edward O. Otis and Vincent G. Bowditch, of Boston; Dr. George Blumer, of Albany; E. V. Stoddard, of Rochester; Edward R. Baldwin, of Saranac Lake, and on the part of the legislature by Senator Horace White, of Syracuse, and Assemblyman Otto Kelsey, of Geneseo. The discussion which followed was spirited, even at times full of ginger, and was participated in by Drs. John H. Pryor and Henry R. Hopkins, of Buffalo; Dr. Daniel Lewis, of New York; Dr. Nelson H. Henry, member of assembly, and others.

On Wednesday morning the members of the Society were given a trolley ride to the new Albany hospital where a clinical session was held under the guidance of Drs. Vander Veer and Macdonald, attending surgeons. Interesting surgical and gynecological cases were presented to the society and the members were conducted through the hospital building. Just before the close of the morning session the president delivered the annual address, taking for his subject, The relation of the clinical laboratory to modern surgery. Dr. Macdonald fully demonstrated the hold which bacteriology has upon modern surgical procedures. In the afternoon two addresses were delivered one by President Raymond, of Union University, on Education and the Practice of medicine, and one by the Rev. Dr. James M. Bulkley, of New York, On Rational Therapeutics versus Christian Science and similar superstitions. Both speakers were accorded a hearty welcome and an attentive hearing. Following the addresses the members were received by Governor Roosevelt in the executive chamber. The banquet which followed at 8 o'clock at the Ten Eyck was well attended and passed off to the greatest satisfaction of all present. Among the guests who attended were Senator Grady, of New York, Speaker Nixon, of Westfield and Assemblyman Kelsey, of Geneseo. Governor Roosevelt, unable to attend, was represented by his private secretary, Mr. Youngs.

The professional papers read were all of scientific worth and evinced a healthy spirit of study and investigation among the individual members. Buffalo and Western New York were well represented on the program and in the appointment of prominent places on the standing committees. A compliment paid the Buffalo delegation was that their speakers were equal if not superior to those of any other section of the state.

The business committee, under the able chairmanship of Dr. Wendell Phillips, of New York, deserves great credit for the excellent program presented and for the dexterous management with which it was so admirably dealt.

The election of officers occurred Thursday morning and resulted as follows: President, A. M. Phelps, of New York; vice-president, George Seymour, of Utica; secretary, Frederic Curtis, of Albany; treasurer, O. D. Ball, of Albany. The standing committees remain as before, with the exception of the addition of A. G. Root, of Albany, and A. E. Davis, of New York to the committee of arrangements; John H. Pryor, of Buffalo, to the committee on hygiene; T. Z. Jones, of Waterville, to the committee on ethics, and O. D. Ball, of Albany, to the committee on publication.

